# Fabrication and characterization of ZnO-NP-doped gelatin/PEG/chitosan composite films for the pharmaceutical and photocatalytic degradation of dyes

**DOI:** 10.1039/d6ra06929k

**Published:** 2026-09-08

**Authors:** Maqam Ali, Muhammad Kaleem Khosa, Awal Noor, Sadaf Qayyum

**Affiliations:** a Department of Chemistry, Government College University Faisalabad Pakistan mkhosapk@yahoo.com; b Department of Chemistry, College of Science, King Faisal University Al-Ahsa Saudi Arabia anoor@kfu.edu.sa

## Abstract

Composite films of gelatin/polyethylene glycol/chitosan (GPC) doped with varying amounts (0.5–2.5% w/w) of zinc oxide nanoparticles (ZnO-NPs) were prepared by a solution casting/ultrasonic method. The ZnO-NPs with average crystallite sizes of 10.20 nm were successfully synthesized by the co-precipitation method, and the formation of the GPC/ZnO-nanocomposite films was confirmed by Fourier-transform infrared (FT-IR) spectroscopy, X-ray diffraction (XRD) and scanning electron microscopy (SEM). UV-vis diffuse reflectance analysis showed a modest decrease in the optical band gap from approximately 3.24 eV for bare ZnO to 3.18 eV for GPC–ZnO containing 2.5% ZnO. FT-IR spectroscopy revealed a bathochromic shift of the amide I, amide II and O–H/N–H stretching bands, which confirmed the strong hydrogen bonding and coordination interactions between the ZnO-NPs and the functional groups of the polymers. The XRD studies showed that the hexagonal wurtzite structure of the ZnO NPs was retained in the amorphous biopolymer matrix with up to 2% loading. SEM studies confirmed the homogeneous dispersion of the NPs in the biopolymer without any agglomeration. Mechanical properties and water barrier (water vapor permeability, moisture absorption and water solubility), antibacterial (*Escherichia coli*, *Staphylococcus aureus*, and *Bacillus subtilis*) and 2,2-diphenyl-1-picrylhydrazyl (DPPH) antioxidant activities were found to be the maximum at 2% ZnO loading. The photocatalytic test results revealed that the GPC–ZnO nanocomposite can degrade Direct Green 6 dye by up to ∼95% at the optimized catalyst dose (0.5 g/100 mL) as compared with ∼68% for bare ZnO-NPs. The efficiency of degradation was favored at pH 5–7 and with increasing temperatures from 30 °C to 50 °C. Based on the biological results and photocatalytic degradation of dyes, the ZnO-NP-doped GPC nanocomposite films reported herein are promising as multifunctional materials for applications such as wound dressing or antimicrobial barrier films and for the photocatalytic treatment of dye-containing water.

## Abstract 2

Composite films of gelatin/polyethylene glycol/chitosan (GPC) doped with varying amounts (0.5–2.5% w/w) of zinc oxide nanoparticles (ZnO-NPs) were prepared by a solution casting/ultrasonic method.

## Graphical abstract

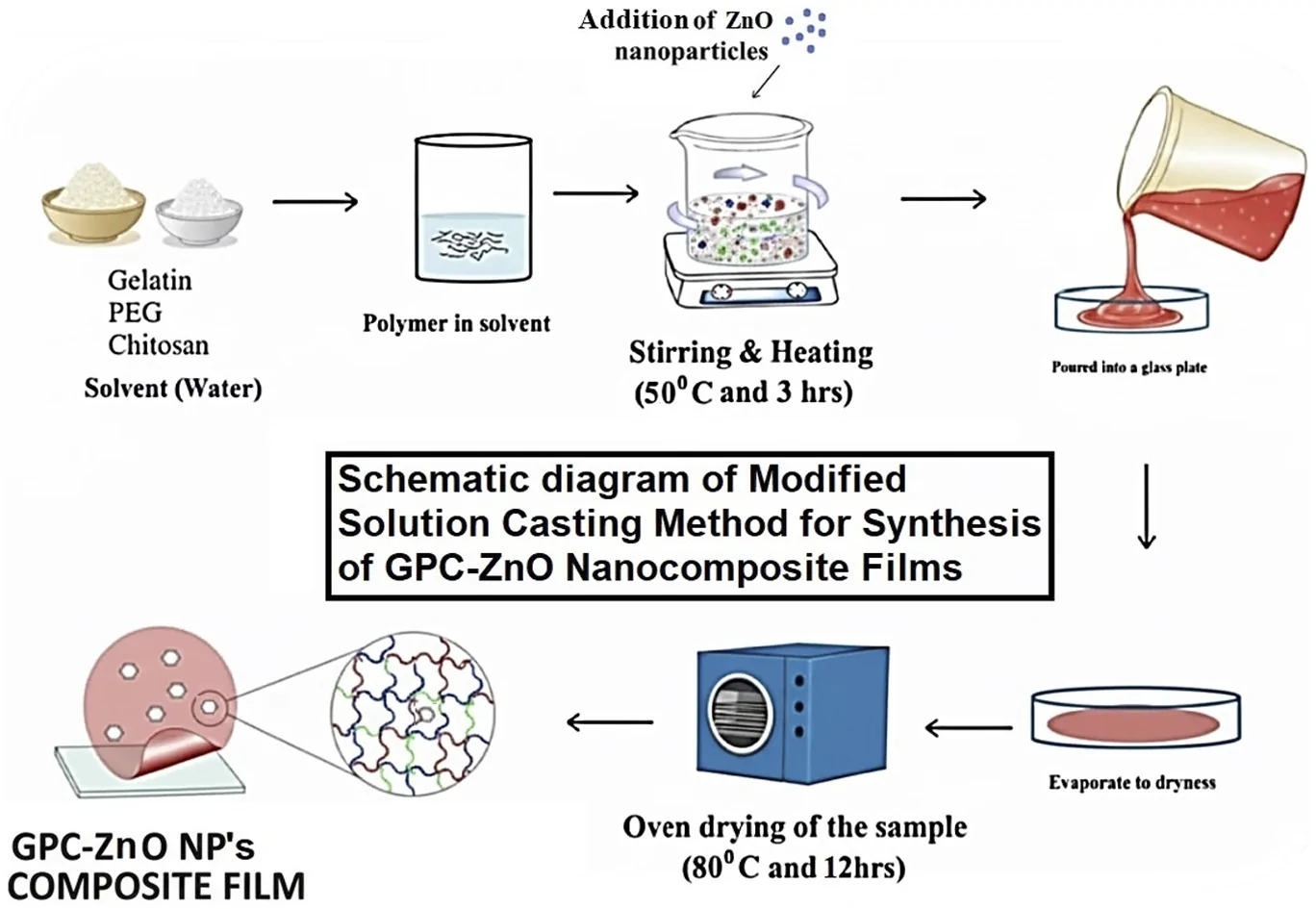

## Introduction

Gelatin, polyethylene glycol (PEG) and chitosan have wide applications in the field of pharmaceutical sciences due to their remarkable biocompatibility, biodegradability, and wound-healing properties and for the formulation of versatile drug-delivery systems.^1^ Gelatin, a natural denatured protein product obtained from collagen, is highly biodegradable and offers good cell affinity, facilitating its use as a matrix for controlled drug-release systems.^2^ It is extensively employed in capsule synthesis, microencapsulation and the formulation of hydrogels as a safe matrix for the controlled release and improved stability of drugs.^3^ Gelatin contains multiple copies of the sequence arginine–glycine–aspartic acid (RGD), which is important for cell adhesion and proliferation and can be used in scaffolds to stimulate tissue regeneration.^4^

Polyethylene glycol (PEG) is a synthetic hydrophilic polymer that is widely employed to modify drug carriers and therapeutic agents to increase their solubility and circulation time and decrease immunogenicity. It is an important excipient in many pharmaceutical formulations, such as for drug solubilization and drug carrier modification (PEGylation), and can enhance the bioavailability of drugs by increasing the circulation time and reducing immunogenicity.^5^ PEG helps by providing a less immunogenic and more hydrophilic environment and promotes the formation of hydrogels, which is good for moisture content, cell infiltration and nutrient diffusion.^6^

A natural polysaccharide product known as chitosan possesses mucoadhesive and permeation–enhancing properties that are particularly attractive for drug delivery systems, such as oral, nasal, ocular and transdermal delivery systems.^7^ In addition to its mucoadhesive property, it possesses bioadhesivity, the ability to open tight epithelial junctions and enhance the permeation of drugs across biological membranes.^8^ Chitosan is also reported to exhibit intrinsic antibacterial activity and haemostatic properties that promote wound contraction by accelerating tissue repair by increasing fibroblast migration and the deposition of collagen.^9^

The combination of zinc oxide nanoparticles (ZnO-NPs) with biocompatible polymers to create multifunctional nanocomposites with enhanced antimicrobial properties, controlled drug delivery, wound-healing ability and excellent biocompatibility is very effective.^10^ The therapeutic ability and minimized side effects achieved by combining these polymers with ZnO-NPs have led to the invention of novel multifunctional drug-delivery systems that have been shown to be therapeutic in modern pharmaceutical sciences, especially in cancer therapy and regenerative medicine.

One of the direct dye group of dyes, Direct Green 6 (DG-6, C.I. 30295), is widely used for dyeing cotton, silk and nylon cloths, since it possesses good affinity for cellulosic fibers and is cost-effective. It is composed of elongated conjugated azo linkages (–N=N–), which give rise to its distinctive green colour; however, these same characteristics lead to the low biodegradability and persistence in aquatic environments of DG-6 and are one of the primary issues for ecotoxicity.^11^

Despite the potential attributes, few comparative studies have been performed to evaluate the effect of the increase in ZnO-NP concentration in GPC composite films on the physicochemical, mechanical, barrier, antibacterial, and antioxidant properties. Thus, the present study is focused on the synthesis and characterization of various concentrations of ZnO-NPs (0.5–2.5% w/w) incorporated in a composite film made up of gelatin/PEG/chitosan (GPC), formed by the solution casting method, to optimize the formulation for photocatalytic and pharmaceutical applications.

## Experimental

### Materials and reagents

All chemicals, including laboratory grade gelatin, type B medical grade chitosan powder (molecular weight 30 kDa and degree of deacetylation 90%), polyethylene glycol (PEG, molecular weight 8000), zinc nitrate hexahydrate [Zn(NO_3_)_2_·6H_2_O], sodium hydroxide (NaOH), nitric acid (HNO_3_), hydrogen peroxide (H_2_O_2_) and Direct Green 6 dye (DG-6), were obtained from Sigma-Aldrich, Pakistan, and used without any further purification.

### Chemical characterization

All major functional groups and band shifts of the synthesized GPC–ZnO nanocomposite films were characterized using an FT-IR spectrophotometer (PerkinElmer, Bruker) in the wavenumber range of 400–4000 cm^−1^. An X-ray diffractometer (Bruker D8 Advance) with Cu Kα radiation (*λ* = 1.5405 Å; 30 kV, 40 mA) was used to investigate the structural phase and crystallinity, with a scanning rate of 5° min^−1^. UV-vis diffuse reflectance spectra were recorded over 300–800 nm (PerkinElmer Lambda 950 UV-vis-NIR Spectrophotometer). Reflectance data were converted using the Kubelka–Munk function *F*(*R*) = (1 − *R*)^2^/(2*R*), and the direct optical band gap was estimated by extrapolating the linear region of [*F*(*R*)*hν*]^2^*versus hν* to the energy axis. The mechanical properties, including Young's modulus, tensile strength and percentage elongation at break, were measured with a DMA Q800 V21.3 Build 96. The surface morphologies were investigated by scanning electron microscopy (SEM) (FEI-NOVA Nano SEM-450).

### Synthesis of zinc oxide nanoparticles

ZnO NPs were prepared using the co-precipitation method with slight modifications.^12^ The average crystallite size obtained from XRD/Scherrer analysis was approximately 10.20 nm. In short, zinc nitrate hexahydrate (2.975 g) was dissolved in deionized (DI) water (100 mL). A solution of NaOH (0.8 g) in DI water (100 mL) was added to it dropwise over a period of 30 minutes with constant stirring. The precipitate obtained was filtered, dried at 90 °C, ground to fine powder, and then calcined in a muffle furnace at 500 °C for 2 hours to obtain a fine powder of white ZnO.

### Synthesis of gelatin/PEG/chitosan composite films

The GPC composite films were prepared using the solution casting technique with slight modification to the reported process.^13^ All the formulations were prepared with the weight ratio of gelatin : PEG : chitosan (2 : 1 : 1). Chitosan was dissolved in 1% (v/v) acetic acid with constant stirring. Gelatin was dissolved in distilled water at 50 °C and PEG in distilled water at room temperature. The three solutions were mixed to create a homogeneous GPC solution. ZnO-NPs were incorporated at 0.5, 1.0, 1.5%, 2.0%, and 2.5% w/w *via* ultrasonic blending. Petri dishes with a 20 cm diameter were cast with each solution and allowed to dry for 12 hours at 80 °C. The film compositions and visual appearances are summarized in Table 1.

**Table 1:** Composition of the gelatin/PEG/chitosan (GPC) composite films with varying ZnO NP contents

Sample	Gelatin (g)	PEG (g)	Chitosan (g)	ZnO (g)	Color	Visual appearance
GPC blend	1.0	0.50	0.50	0	Transparent yellow	Transparent, no bubbles
Blend: 0.5% ZnO-NPs	0.95	0.475	0.475	0.1	Very light yellow	Translucent, no cracks
Blend: 1.0% ZnO-NPs	0.90	0.450	0.450	0.2	Light yellow	Smooth, no cracks
Blend: 1.5% ZnO-NPs	0.85	0.425	0.425	0.3	Very light whitish yellow	Glossy, no cracks
Blend: 2.0% ZnO-NPs	0.80	0.400	0.400	0.4	Whitish yellow	Smooth and glossy, no cracks
Blend: 2.5% ZnO-NPs	0.75	0.375	0.375	0.5	Increased whitish shade	Rough, visible cracks

### Water resistance properties

The water resistance properties of the GPC–ZnO NP composite films were assessed using water vapor permeability (WVP), moisture absorption (MA), and water solubility (WS) by following the standard procedures.^1,2^ All tests were conducted in triplicate.

### Water vapor permeability (WVP)

Pre-dried (70 °C, 12 h) film samples (3.5 × 3.5 cm^2^) were mounted over glass vials containing 20 mL distilled water and incubated at 50 °C. Weight loss over seven days was recorded. The water vapor transmission rate (WVTR) was calculated as follows:
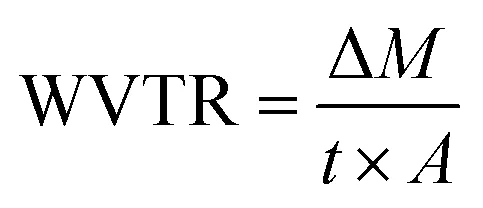
where Δ*M* is the change in weight (g), *t* is time (days), and *A* is the exposed film area (m^2^). WVP (g mm per m^2^ per day per kPa) was subsequently calculated as follows:
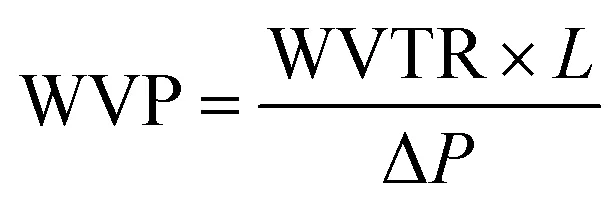
where *L* is film thickness (mm), and Δ*P* is the water vapor pressure difference (12.344 kPa at 50 °C).

### Moisture absorption (MA)

Dried film samples (2 × 2 cm^2^) were equilibrated at 0% RH in a desiccator with dehydrated silica gel (initial weight *W*_0_), then transferred to a desiccator containing saturated calcium nitrate solution (55% RH, 25 °C). Samples were reweighed daily for seven days. MA was calculated according to the following equation.
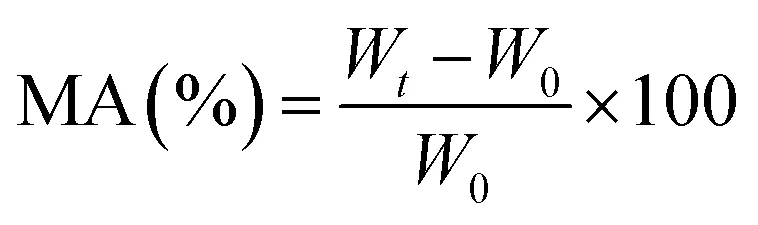


### Water solubility (WS)

Film samples (3 × 3 cm^2^) were dried at 100 °C, weighed (*m*_0_), immersed in distilled water for 24 h, and re-dried to obtain final weight (*m*_1_). WS was calculated as
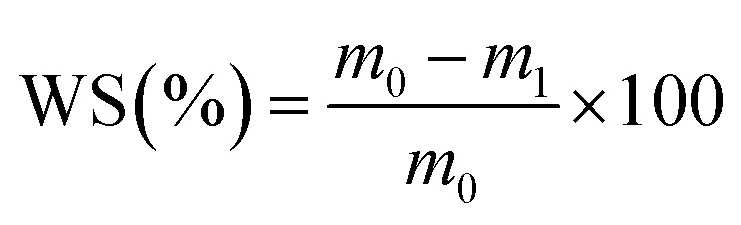


### Biological activities

#### Antibacterial assay

The antibacterial activity was determined by the disc diffusion method using *Escherichia coli* (ATCC-8739), *Staphylococcus aureus* (ATCC-6538) and *Bacillus subtilis* (ATCC-6633). Film samples were dissolved in dimethyl sulfoxide (DMSO) at 20 mg mL^−1^ and were then transferred onto Mueller–Hinton agar plates prepared with bacterial suspension (106 CFU mL^−1^). The plates were then incubated at 37 °C for 24 h, and the inhibition zone diameters (mm) were measured. All experiments were repeated three times. Cefixime was used as a positive control.

#### Antioxidant assay

The free radical scavenging potential was determined by the DPPH (2,2-diphenyl-1-picrylhydrazyl) assay (with slight modifications).^15^ A stock solution of film was prepared at 5 mg mL^−1^ in DMSO and tested at 20, 40, 60, 80, 100 and 200 µg mL^−1^ 20 µL of each concentration was mixed with 2980 µL of DPPH (0.1 mM) in methanol, incubated at 37 °C for 30 min in dark conditions, and the absorbance was later measured at 517 nm. Ascorbic acid (5 mg mL^−1^) was used as a reference antioxidant. The DPPH scavenging activity (%) was calculated according to the following equations.
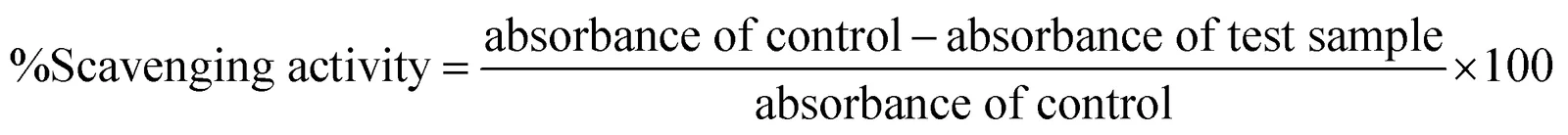


All experiments were conducted in triplicate, and IC_50_ values were determined by nonlinear regression.

#### Photocatalytic degradation experiments

The photocatalytic degradation experiments were performed in a cylindrical borosilicate glass reactor with a 100 W tungsten–halogen light source, which was placed 10 cm from the reactor and emitted visible light. The photocatalysts of fixed amount (0.1 g–0.9 g/100 mL) were ultrasonicated in 100 mL of Direct Green 6 aqueous solution (initial concentration 10 mg/100 mL). Before irradiation, the suspension was shaken in the dark for 30 minutes to attain the adsorption–desorption equilibrium. The samples were then subjected to irradiation, and the remaining concentration of DG-6 was monitored at different time intervals during the irradiation process (0, 10, 20, 30, 40, 50, 60, 70, 80 and 90 minutes) by removing the catalyst from the solution through centrifugation and measuring it using the UV-vis spectrometer at *λ*_max_ ≈ 623 nm.

The degradation efficiency (%) was calculated as follows:
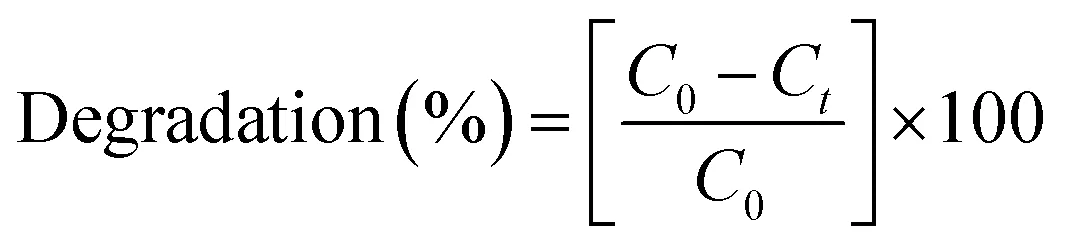
where *C*_0_ is the initial DG-6 concentration, and *C*_*t*_ is the concentration at time *t*. The effects of key parameters, including initial dye concentration (10–50 mg/100 mL), catalyst dosage (0.1–0.9 mg/100 mL), and solution pH (3–11), were investigated. Scavenging experiments were performed by adding isopropanol (IPA, ˙OH scavenger), benzoquinone (BQ, ˙O_2_^−^ scavenger), and ethylenediaminetetraacetic acid disodium salt (EDTA–Na_2_, h^+^ scavenger), each at a concentration of 1 mmol L^−1^, to identify the principal reactive species.

## Results and discussion

### FTIR analysis

**Fig. 1:**
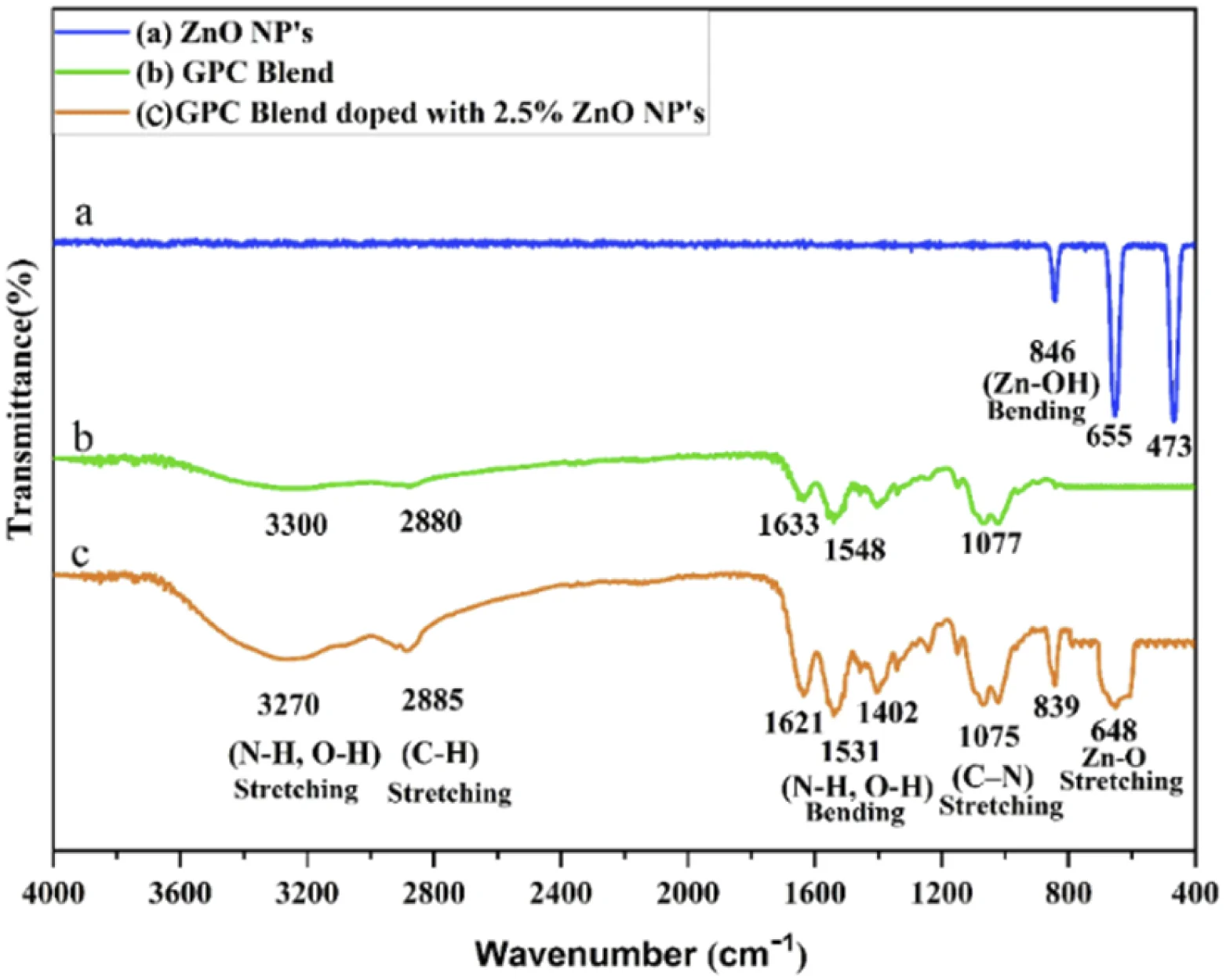
FTIR spectra of the (a) ZnO nanoparticles, (b) gelatin/PEG/chitosan (GPC) blend, and (c) GPC blend doped with 2.5% ZnO-NPs.

FTIR spectra of the pure ZnO-NPs, GPC blend and GPC doped with 2.5 wt% of ZnO-NPs are shown in Fig. 1.

The absorption spectra of pure ZnO-NPs (Fig. 1a) showed absorption bands at 846, 655 and 473 cm^−1^, which were identified as the Zn–OH bending and the Zn–O stretching vibration, respectively, indicating successful formation of the NPs. The pristine GPC blend (Fig. 1b) exhibited a wide absorption band around 3300 cm^−1^ due to the overlapping stretching vibrations of O–H and N–H of the constituents of GPC (gelatin, chitosan and PEG), and a C–H stretching band at 2880 cm^−1^. The appearances of amide I (C=O stretching) and amide II (N–H bending) bands at 1633 and 1548 cm^−1^, respectively, and the presence of a band at 1077 cm^−1^ are attributed to the C–O/C–N stretching band, proving the successful polymer blending process by intermolecular hydrogen-bonding interactions.^14^ The stretching band of O–H/N–H decreased from 3300 to 3270 cm^−1^, the amide I band shifted to 1621 cm^−1^, and the amide II band shifted to 1531 cm^−1^ upon the incorporation of 2.5 wt% ZnO-NPs (Fig. 1c). The Zn–O stretching band was observed at 648 cm^−1^, which is indicative of ZnO integration. Bathochromic shifts of these bands indicate strong intermolecular interactions of the ZnO-NPs with the polymer functional groups, especially the hydrogen bonding and coordination interactions of the hydroxyl, amino and carbonyl functional groups, indicating good dispersion of the NPs and formation of a stable polymer–nanoparticle network. The same changes in the FTIR spectra of gelatin/chitosan/ZnO nanocomposites have been reported previously.^16^

The FTIR data show that the ZnO-NPs were successfully synthesized and incorporated into the GPC polymer. The bathochromic shifts of the amide I, amide II and O–H/N–H stretching bands observed in the nanocomposite films are indicative of strong interfacial H-bonding and coordination interactions between surface hydroxyl groups of ZnO and the functional groups of gelatin and chitosan. Such interactions play a key role in providing uniformity of dispersion of the nanoparticles and in maintaining the integrity of the composite, as observed for other related gelatin/chitosan/ZnO systems.

### XRD analysis

**Fig. 2:**
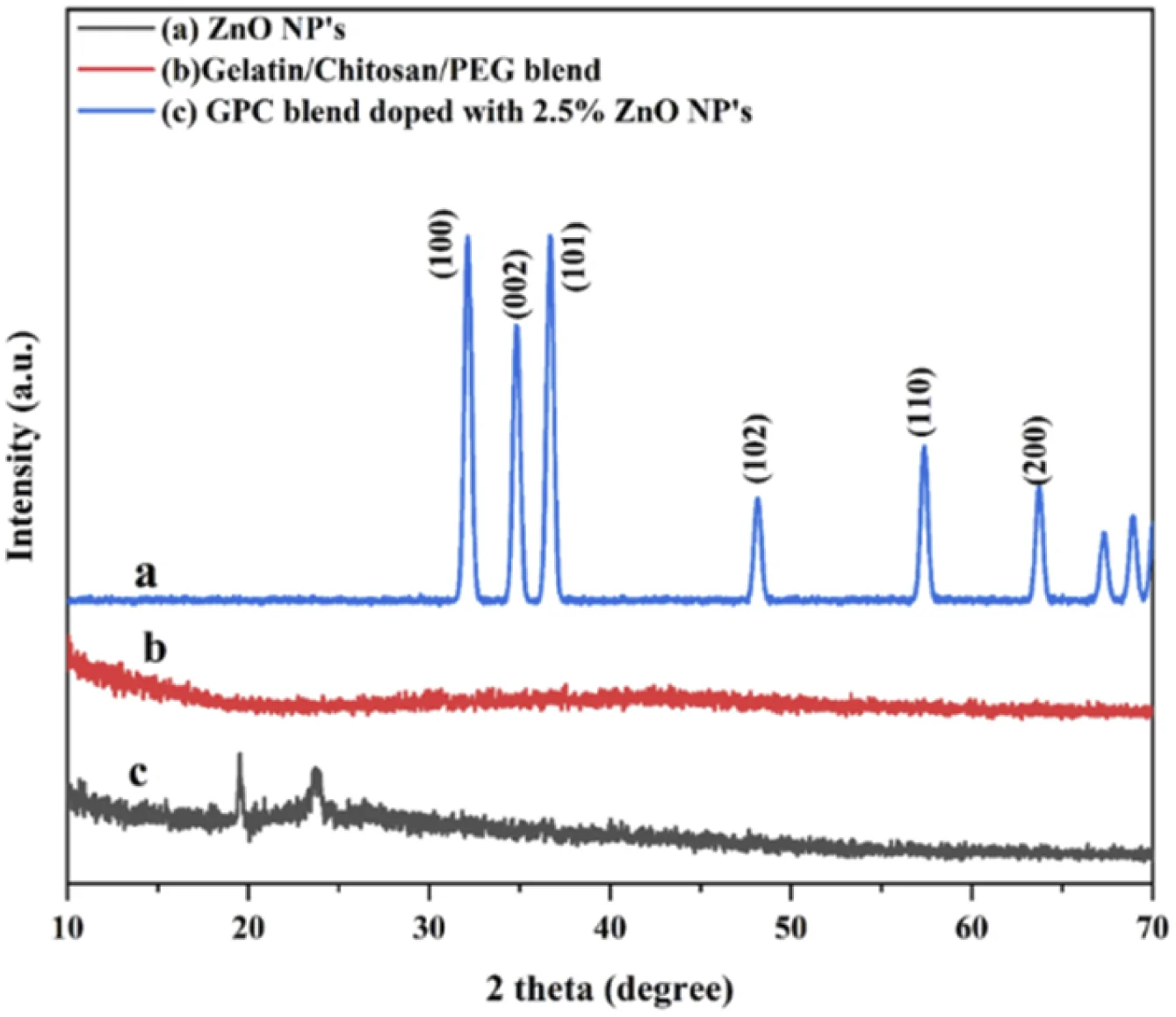
XRD patterns of the (a) ZnO-NPs, (b) GPC blend, and (c) GPC blend doped with 2.5% ZnO-NPs.

The XRD pattern of pure zinc oxide nanoparticles (ZnO-NPs), GPC blend and GPC doped with 2.5 wt% of ZnO-NPs are shown in Fig. 2. Pure ZnO-NPs (Fig. 2a) displayed sharp diffraction peaks at 2*θ* ≈ 32.0°, 34.5°, 36.3°, 47.5°, 56.6°, and 62.9°, corresponding to the (100), (002), (101), (102), (110), and (200) crystallographic planes of the hexagonal wurtzite structure (JCPDS card no. 36-1451), confirming phase purity and high crystallinity.^17^

The neat GPC blend (Fig. 2b) showed a broad diffuse halo in the range of 15–25°, which is indicative of the amorphous nature of the polymer matrix present. It was found that the characteristic peaks of ZnO were observed in addition to the halo of amorphous polymer in samples with 2.5 wt% ZnO-NP added (Fig. 2c), which implies that the crystallinity of the ZnO was maintained in the polymer matrix. The reduced intensities of the peaks compared to pure ZnO-NPs suggest a low content of filler, indicating that the NPs were embedded in the polymer. There were no other peaks present corresponding to impurities, which shows the good compatibility of ZnO–GPC and the formation of a semi-crystalline nanocomposite system.

The crystallinity of the wurtzite ZnO was retained in the amorphous biopolymer matrix, as confirmed by XRD, which resulted in the formation of semi-crystalline nanobiocomposites. The intensities of the peaks of the obtained ZnO-NPs decreased compared to pure ZnO-NPs, indicating low filler concentration and encapsulation in the polymer chains, and no extra impurity peaks were observed, indicating good compatibility between ZnO-NPs and GPC. The broad amorphous halo of the GPC matrix and the presence of the characteristic diffraction peaks of ZnO further confirm the uniform dispersion of the crystalline NPs in the polymer matrix, which is essential for uniform functional performance. The average crystallite size of the ZnO NPs was calculated by using the Debye–Scherrer equation and found to be 10.20 nm, as shown in Table 2.

**Table 2:** X-ray diffraction peak positions of ZnO NPs^a^

Peak position 2*θ* (°)	FWHM *β* (°)	Crystallite size (nm)
31.77	1.5065	5.73
34.42	1.1342	7.66
36.25	1.9069	4.58
47.54	0.4685	19.35
56.6	0.7091	13.29
62.86	1.0819	8.99
66.38	0.7553	13.13
67.96	1.1261	8.89
Average crystallite size (nm)		10.20 nm

a Abbreviation: FWHM—full-width at half maximum.

### UV-vis diffuse reflectance and optical band-gap analysis

**Fig. 3:**
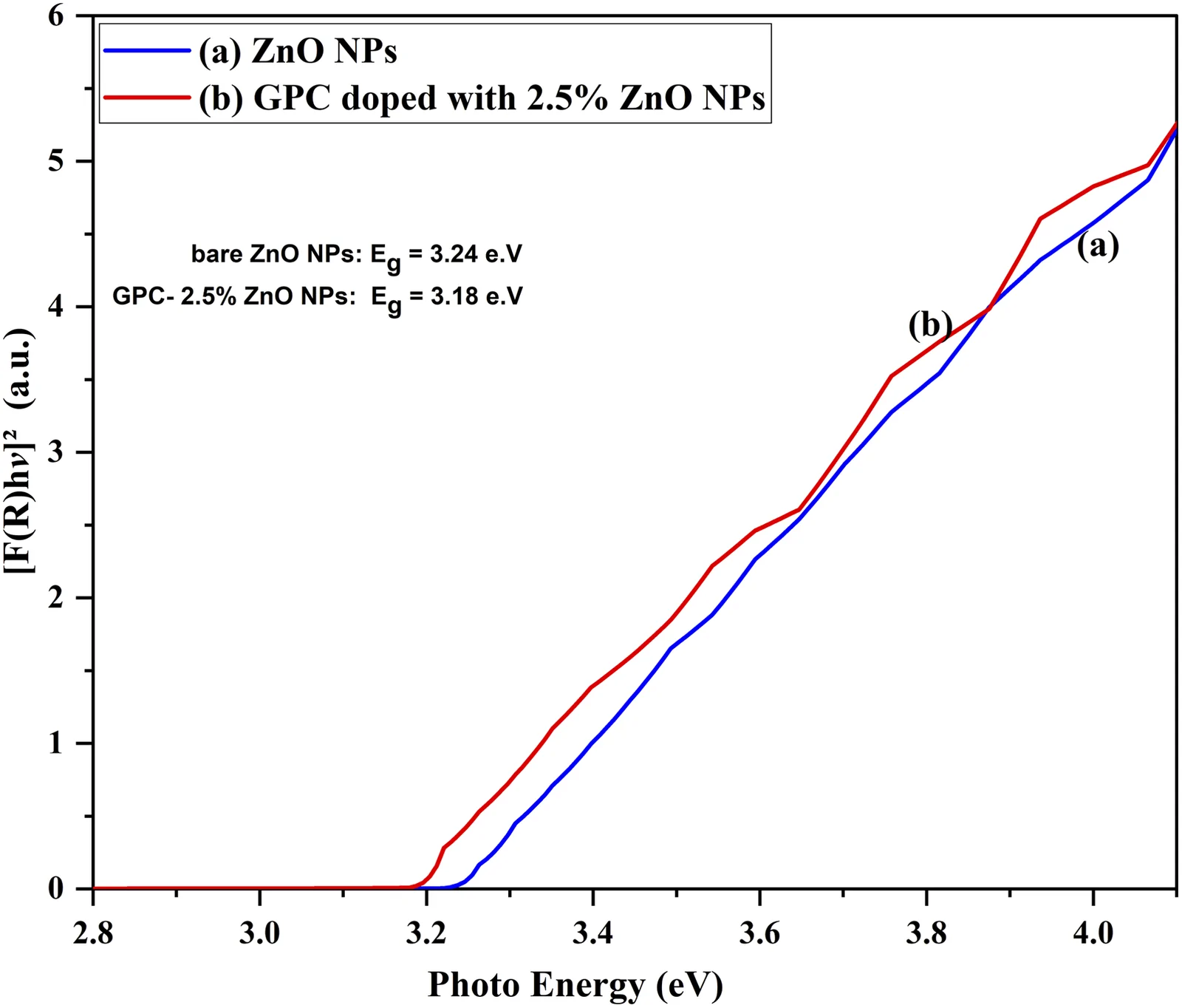
Tauc plots derived from the UV-vis diffuse reflectance data of (a) bare ZnO and (b) GPC–ZnO containing 2.5% ZnO.

The optical response of bare ZnO and optimized GPC–ZnO film (2.5% ZnO) was investigated using UV-vis diffuse reflectance spectroscopy. The Kubelka–Munk-transformed Tauc plots for a direct transition are shown in Fig. 3. These were obtained by linear extrapolation of the [*F*(*R*)*hν*]^2^ to the photon-energy axis, which resulted in optical band-gap values of about 3.24 eV for the bare ZnO and 3.18 eV for GPC–ZnO. The absorption edge is slightly redshifted with incorporation of ZnO into the GPC matrix, with a small decrease of ∼0.06 eV. The shift is rather small; thus, enhanced photocatalytic activity of GPC–ZnO is attributed to the combination of this optical modification, ZnO dispersion, dye adsorption on the polymer surface, and reduced charge-carrier recombination, rather than only band-gap narrowing.^21^

### SEM morphology

**Fig. 4:**
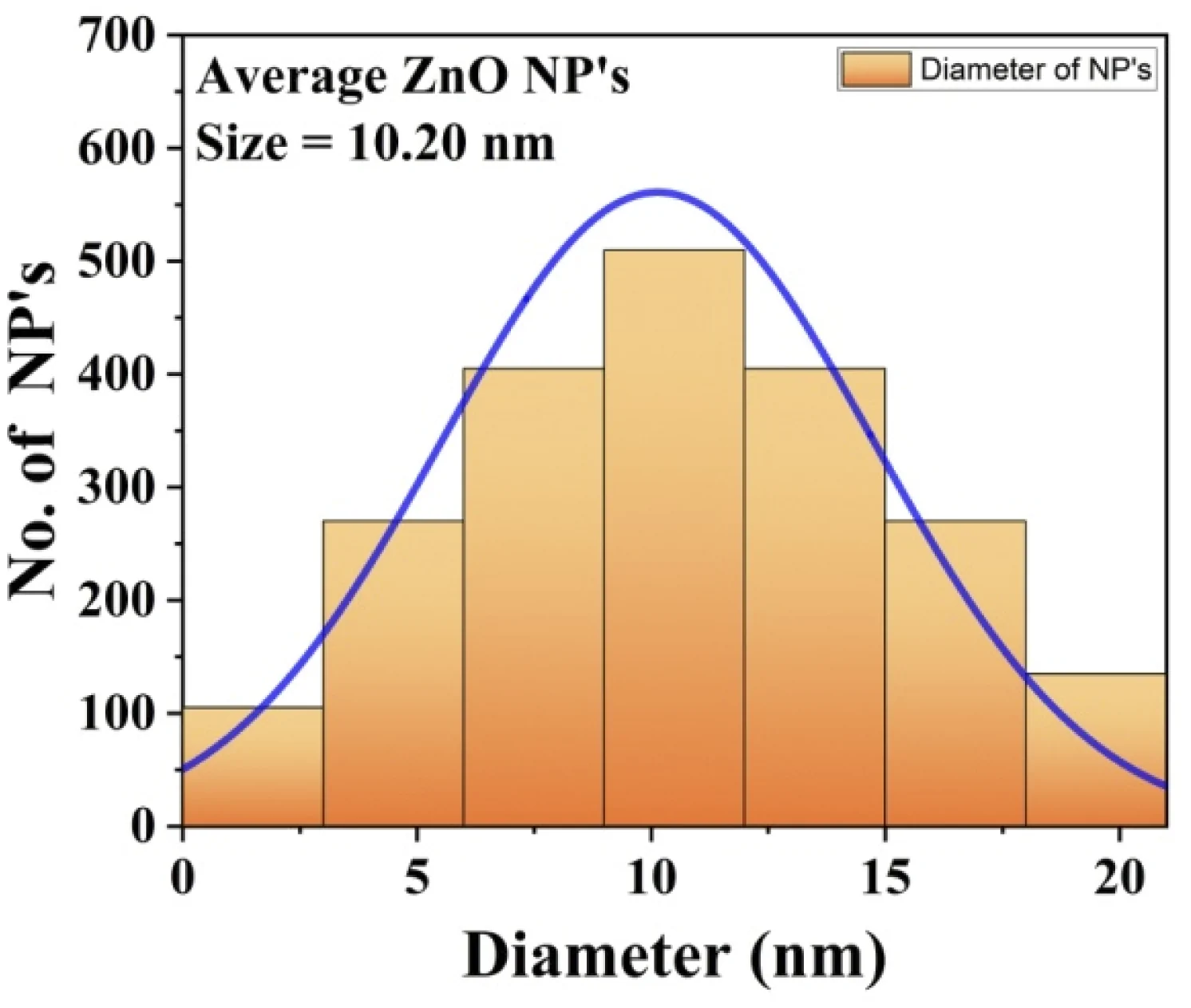
Particle-size distribution of ZnO-NPs obtained from SEM image analysis. The value of 10.20 nm represents the average size and not a uniform particle diameter.

**Fig. 5:**
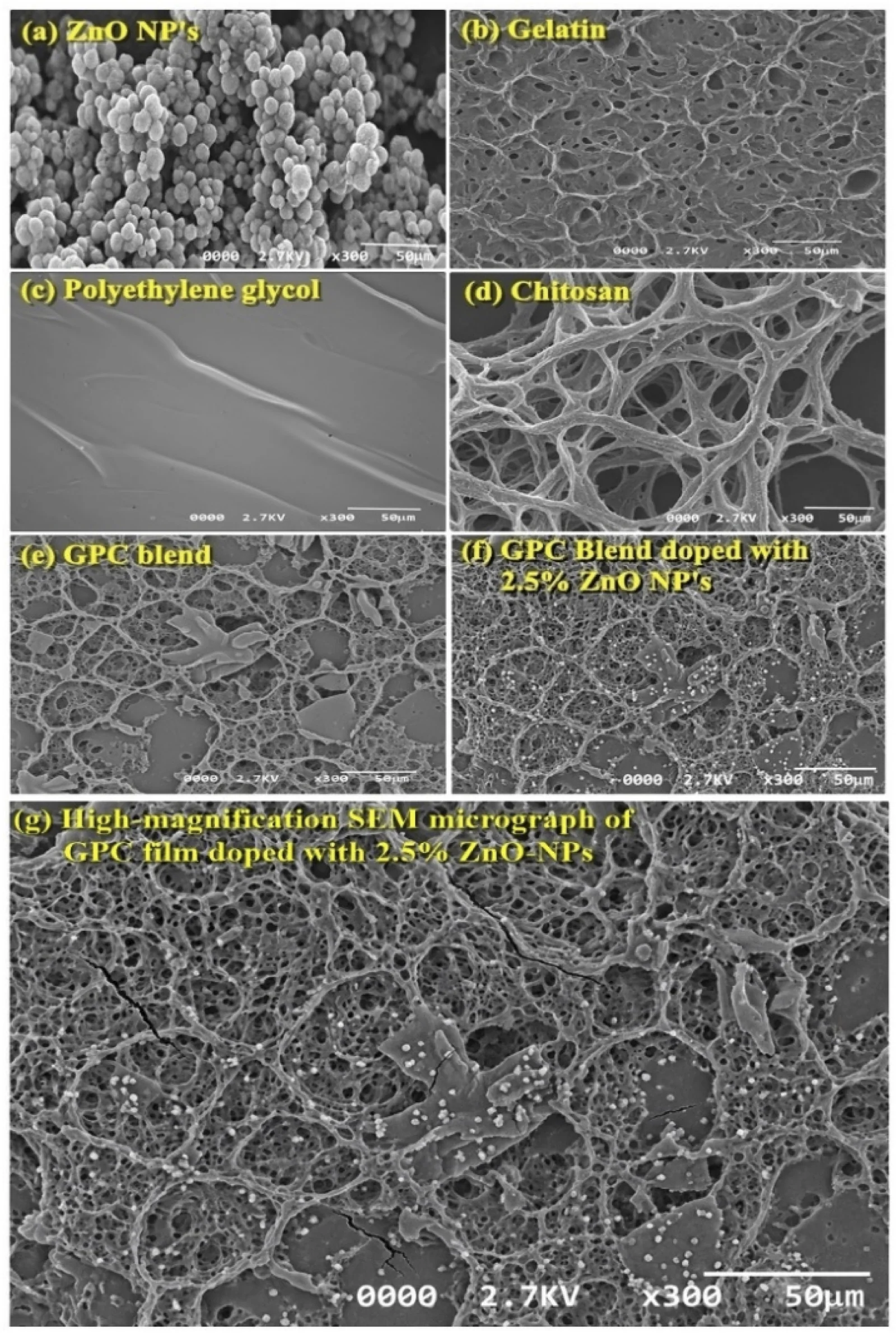
SEM images of (a) ZnO-NPs, (b) gelatin, (c) PEG, (d) chitosan, (e) GPC blend, and (f) GPC blend doped with 2.5% ZnO-NPs. (g) High-magnification SEM micrograph of the GPC film containing 2.5% ZnO-NPs, showing localized crack formation and surface defects.

A histogram based on SEM images also confirmed that the average size distribution of ZnO-NPs was 10.20 nm (Fig. 4). SEM images of the films are shown in Fig. 5. The ZnO-NPs (Fig. 5a) exhhibited a spheroidal morphology with relatively uniform nanoscale dimensions. The image of pure gelatin (Fig. 5b) revealed a highly porous network structure with more interconnectedness, which is a characteristic of a hydrophilic polymer matrix. The surface of the PEG (Fig. 5c) was smooth, homogeneous and non-porous, as it is a plasticizing material. Chitosan (Fig. 5d), on the other hand, had a fibril-like web-like matrix, characteristic of a semi-crystalline polysaccharide, and exhibited an open, interconnected porous network.

The GPC composite film (Fig. 5e) showed a more uniform and integrated porous morphology than the individual components, which indicated the successful polymer–polymer interactions. Of particular interest, for the 2.5% ZnO-doped GPC film (Fig. 5f), the well-dispersed spherical NPs were homogeneously distributed within the polymeric film, without apparent agglomeration or phase separation, demonstrating the successful integration of the ZnO NPs. The high-magnification micrograph (Fig. 5g) shows the formation of local cracks in the 2.5% ZnO film, which agrees with the visual observation noted in Table 1. The combination of the SEM images suggests that the dispersion of the ZnO is relatively uniform at lower-to-optimal loading levels, while the 2.5% loading level SEM image suggests that there are localized defects, which may indicate overloading in the formulation. This localized cracking/aggregation can create stress concentration points and voids between the interfaces, which can provide a morphological explanation for the mechanical and barrier performance degradation beyond the optimum 2% loading. This uniform dispersion is expected to enable uniform nanoparticle-mediated functionalities, such as antimicrobial activity, mechanical reinforcement and controlled drug release, and the porous structure is expected to sustain cell adhesion, moisture permeation, and sustained drug diffusion.

The SEM analysis showed that ZnO-NPs were well dispersed in the GPC matrix up to 2%, with no signs of agglomeration, which is crucial to achieve uniform functional performance of the film. The gradual transformation of the morphologies of the individual component microstructures to a unified composite matrix is helpful for the formation of a well-blended nanocomposite system. The porosity of the composite is retained, which is highly beneficial for pharmaceutical applications because it allows cells to adhere to the film and moisture to permeate, thus stabilizing the diffusion of the drug. The uniform dispersion of the ZnO ensures nanoparticle-mediated antimicrobial activity and mechanical reinforcement throughout the film.

### Mechanical properties

**Fig. 6:**
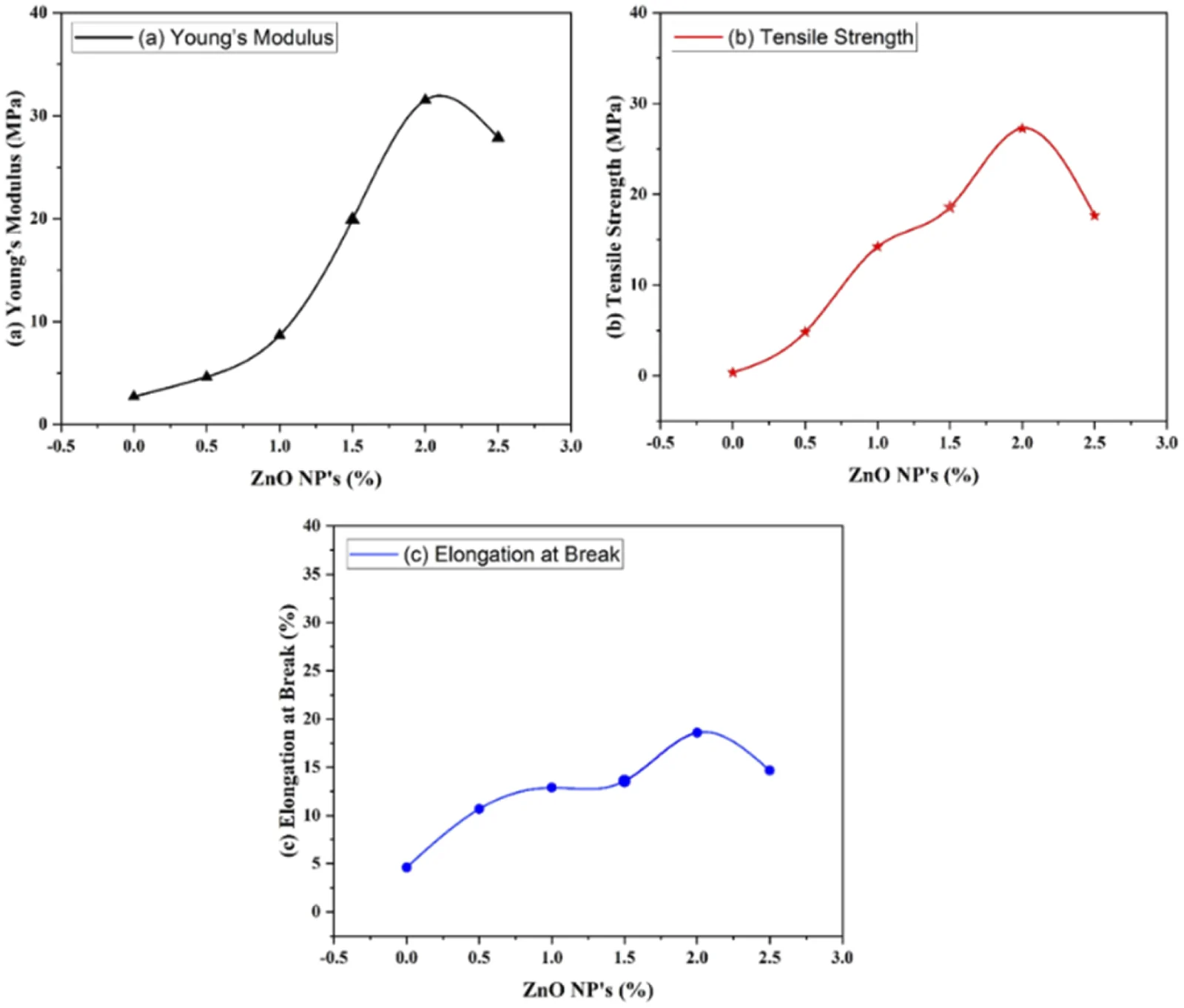
Mechanical properties of the GPC blend doped with varying ZnO-NP concentrations: (a) Young's modulus, (b) tensile strength, (c) elongation at break.

The mechanical properties of the GPC–ZnO composite films are presented in Table 3. The tensile strength (0.37 MPa) and elongation at break (4.62%) of the neat GPC blend were the lowest, as shown in Fig. 6. The Young's modulus and tensile strength increased significantly with the increase in concentration of ZnO-NPs and were maximum at 2% ZnO (tensile strength 27.27 MPa and elongation at break 18.58%). The mechanical performance decreased at 2.5% ZnO-NPs content due to the high content of the NPs, which led to aggregation of the NPs, poor compatibility of the polymer and imbalances in the polymer structure. The increase in mechanical properties with ZnO content up to 2% is attributed to the effective stress transfer between the polymer matrix and the stiff ZnO-NPs due to strong interactions at the interface and the cross-linking of the polymer chains by the NPs, which inhibit the mobility of the polymer chains. The observed decrease with 2.5% ZnO indicates that the NPs are prone to agglomeration and the concentration of stress at the interfaces of clusters causes the generation of micro-defects and a reduction in the reinforcement efficiency. These trends mirror the previously reported mechanical behaviour of biopolymer/ZnO nanocomposites.^18^ The highest tensile strength (27.27 MPa) and the elongation at break (18.58%) at 2% ZnO formulation confirm the optimized loading value for balanced reinforcement and integrity of the polymer network in this GPC system.

**Table 3:** Mechanical properties of the GPC composite films doped with varying ZnO-NP concentrations

Component	Young's modulus (MPa)	Tensile strength (MPa)	Elongation at break (%)
GPC blend	2.700	0.366	4.622
Blend: 0.5% ZnO-NPs	4.630	4.820	10.666
Blend: 1.0% ZnO-NPs	8.666	14.225	12.882
Blend: 1.5% ZnO-NPs	19.902	18.594	13.568
Blend: 2.0% ZnO-NPs	31.531	27.267	18.578
Blend: 2.5% ZnO-NPs	27.863	17.663	14.675

### Water resistance properties

**Fig. 7:**
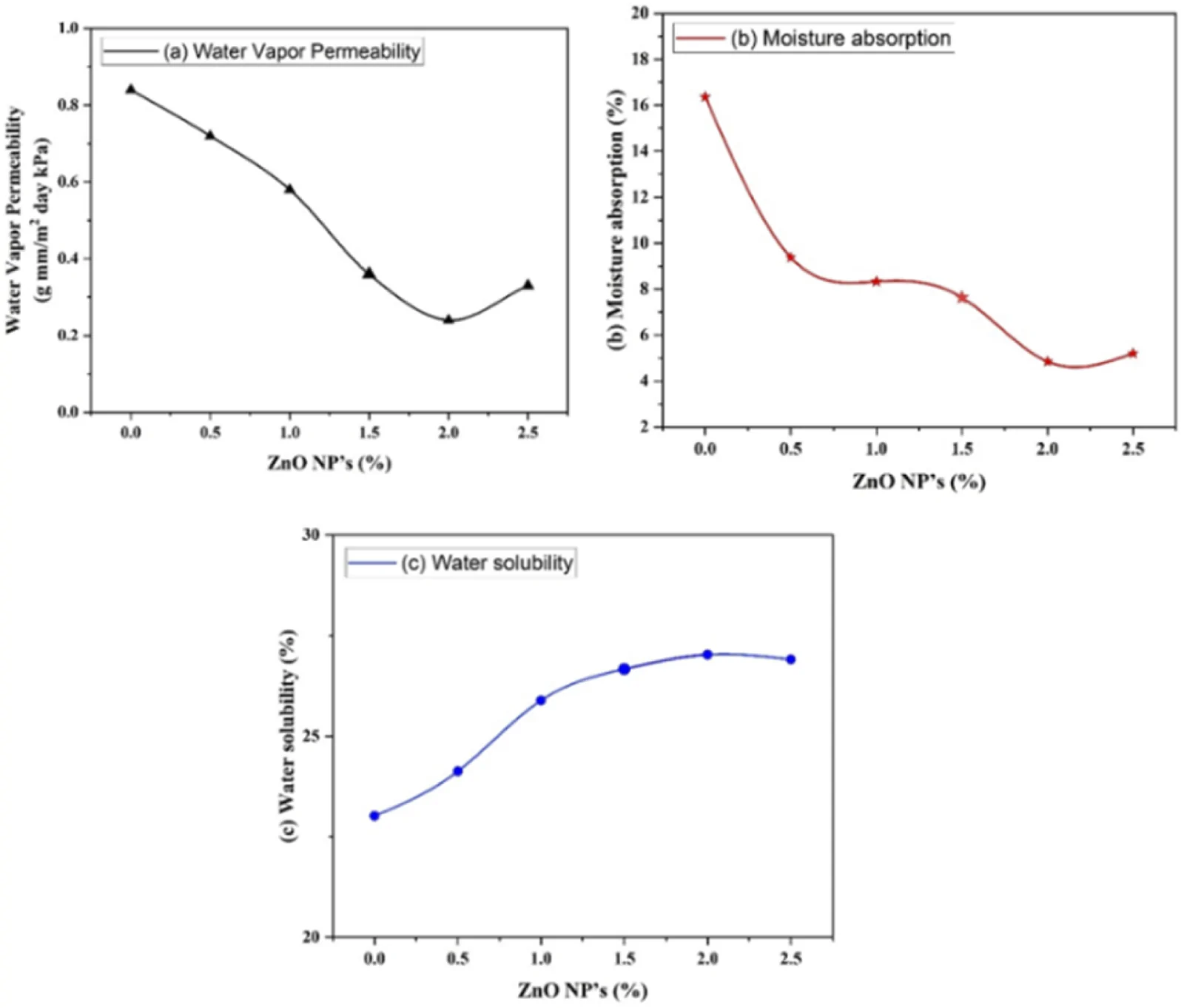
Water resistance properties of the GPC blend doped with varying ZnO NP concentrations: (a) WVP, (b) moisture absorption, and (c) water solubility.

The water resistance properties of films are shown in Fig. 7, and the data on water resistance are given in Table 4. Neat GPC film had the highest WVP (0.84 g mm per m^2^ per day per kPa) and MA (16.36%) values. However, as the concentration of ZnO-NPs increased, the barrier properties improved significantly, with the lowest WVP (0.24) and MA (4.85%) at 2% ZnO, thus indicating that the films were more compact and water uptake was reduced. The addition of ZnO at levels up to 2% showed a slight increase in water solubility, which then remained constant. WVP and MA increased slightly with the increase in filler loading (compared with 2% and 2.5% ZnO samples), signifying poor structural compactness at higher filler loadings. The enhanced water barrier properties after the addition of ZnO are explained by the formation of tortuous diffusion pathways for water molecules in the nanocomposite matrices and crosslinking mediated by the addition of ZnO, which decreases the free volume and increases the compactness of the matrices. It is interesting to note that the slight increase in WVP and MA for 2.5% ZnO is in line with the agglomeration of the NPs, which creates voids at the interfaces allowing the ingress of moisture and hence confirms the optimum amount of ZnO addition at 2% for better barrier properties.^19^ The enhanced barrier performance at 2% ZnO content is notable for pharmaceutical packaging and wound dressing, where excluding moisture is essential for the stability of the drug and for avoiding microbial contamination.

**Table 4:** Water resistance properties of the GPC composite films doped with varying ZnO-NP concentrations

Component	WVP (g mm per m^2^ per day per kPa)	Moisture absorption (%)	Water solubility (%)	Thickness (mm)
GPC blend	0.84	16.36	23.02	0.05
Blend: 0.5% ZnO-NPs	0.72	9.38	24.13	0.05
Blend: 1.0% ZnO-NPs	0.58	8.33	25.89	0.05
Blend: 1.5% ZnO-NPs	0.36	7.64	26.67	0.05
Blend: 2.0% ZnO-NPs	0.24	4.85	27.03	0.05
Blend: 2.5% ZnO-NPs	0.33	5.21	26.91	0.05

### Antibacterial activity

**Fig. 8:**
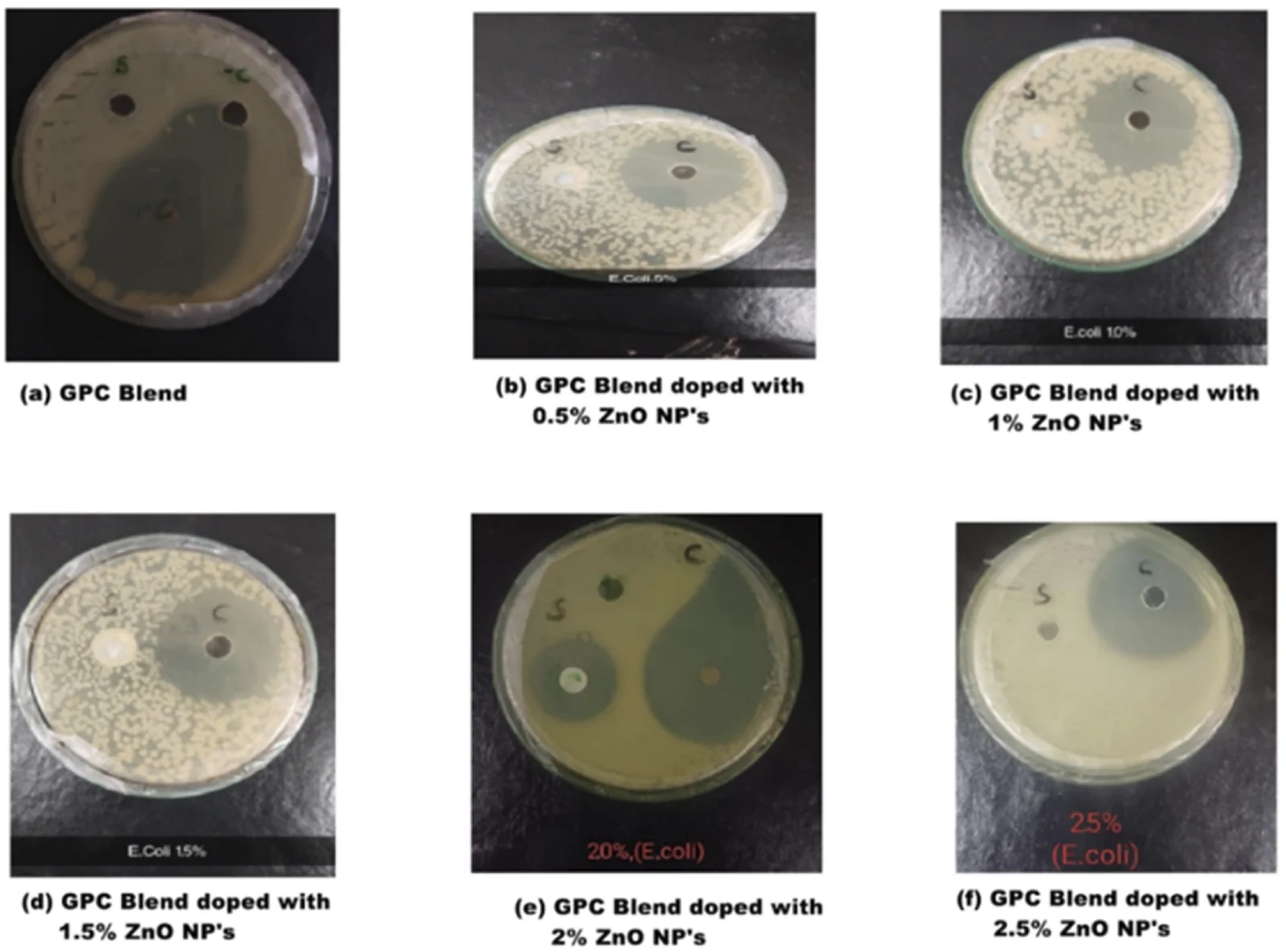
Antibacterial activity against *E. coli* of the (a) GPC blend, (b) 0.5% ZnO-NPs, (c) 1% ZnO-NPs, (d) 1.5% ZnO-NPs, (e) 2% ZnO-NPs, and (f) 2.5% ZnO-NPs.

Table 5 shows the results of antibacterial action studies. The neat GPC film showed low antibacterial activity towards all the strains. The incorporation of the ZnO-NPs gradually improved the antimicrobial activity against *E. coli*, *S. aureus* and *B. subtilis*, with the highest inhibition zone observed for samples with 2% ZnO (27.88 mm, 21.66 mm, and 26.66 mm, respectively), showing strong antimicrobial activity (26–40 mm zone) (Fig. 8). With a higher concentration of NPs (2.5% ZnO), the inhibition zones were significantly reduced for all strains, showing diminished antimicrobial activity, likely due to agglomeration effects. The antibacterial activity of ZnO-NPs is mostly related to the generation of reactive oxygen species (ROS), disruption of the bacterial cell membrane, and the release of zinc ions (Zn^2+^), which interfere with bacterial enzyme activities. The trend of progressive increase in the zones of inhibition with increasing concentration of ZnO (up to 2%) is in line with the concentration-dependent ROS generation and zinc ion release. At 2.5% ZnO, the decreased antibacterial activity might be explained by agglomeration of NPs, which decreases the effective surface area for ROS generation and Zn^2+^ release, and possibly by protection of the denser polymer matrix. These observations are consistent with the reported antibacterial behavior of the biopolymer nanocomposites with ZnO.^10^ The large inhibition zones obtained for both Gram-negative (*E. coli*) and Gram-positive (*S. aureus* and *B. subtilis*) bacteria at 2% ZnO level indicate broad-spectrum antimicrobial activity, which is essential for wound dressing and antimicrobial packaging applications.

**Table 5:** Antibacterial activity (zone of inhibition, mm) of the GPC composite films against selected bacterial strains

Component	*E. coli* (ATCC-8739)	*S. aureus* (ATCC-6538)	*B. subtilis* (ATCC-6633)
GPC blend	1.17	2.66	2.05
Blend: 0.5% ZnO-NPs	18.16	13.44	15.33
Blend: 1.0% ZnO-NPs	22.33	21.33	26.00
Blend: 1.5% ZnO-NPs	24.66	23.41	26.33
Blend: 2.0% ZnO-NPs	27.88	21.66	26.66
Blend: 2.5% ZnO-NPs	10.33	18.09	9.33
Cefixime (standard)	33.15	31.4	29.5

### Antioxidant activity

**Fig. 9:**
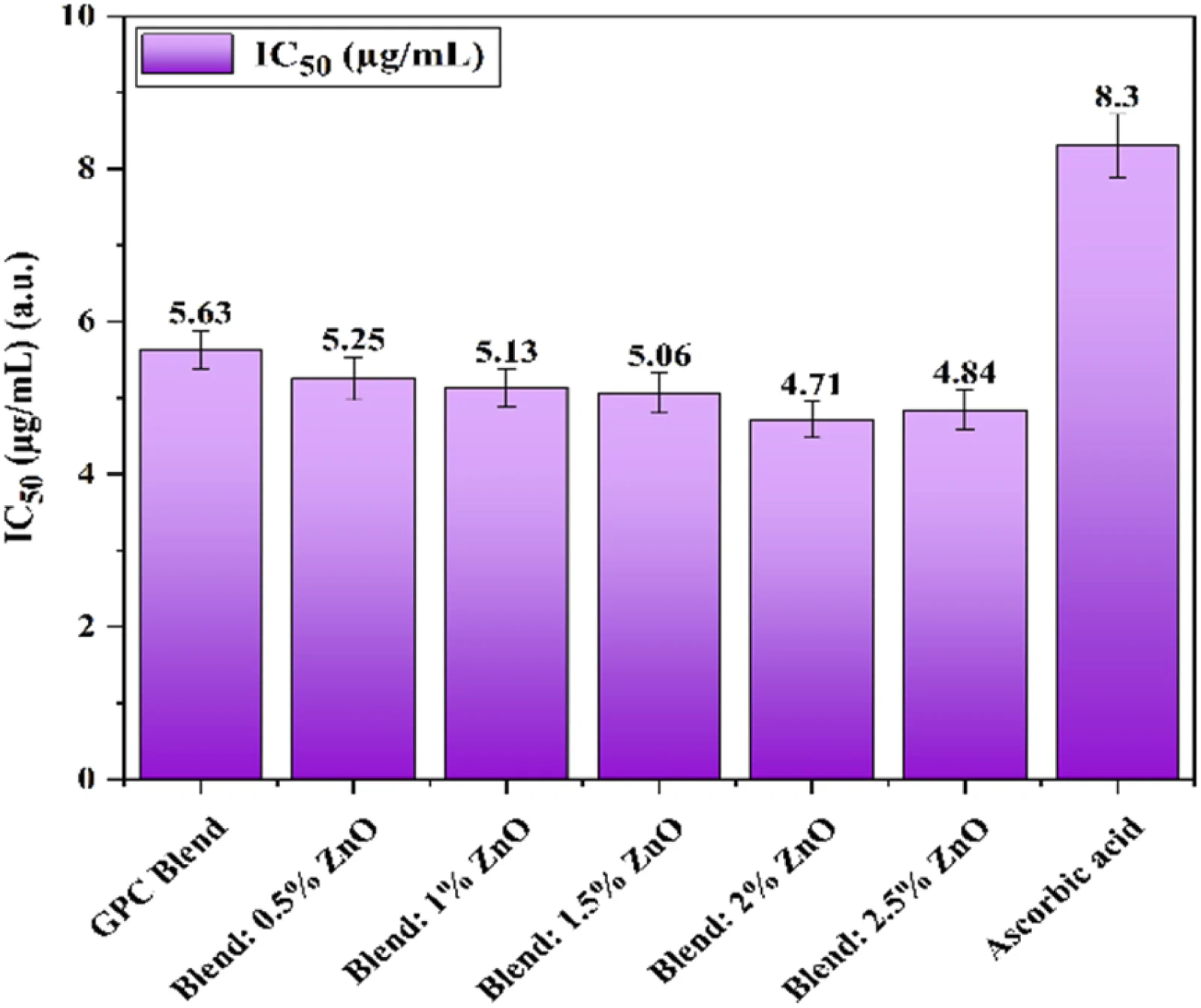
Comparison of the IC_50_ values from the DPPH assay of the neat GPC blend, ZnO-NP-loaded GPC films and ascorbic acid. A lower IC_50_ value indicates stronger radical-scavenging activity.

The results of DPPH free radical scavenging studies are presented in Table 6. Radical scavenging activity was observed to be concentration-dependent for all the formulations. The 2.0% ZnO-doped film had the lowest IC_50_ value of 4.71 µg mL^−1^, which showed the highest antioxidant potential among all the formulations compared to ascorbic acid (IC_50_: 8.30 µg mL^−1^). The IC_50_ values followed the order: blend: 2% ZnO (4.71) < blend: 2.5% ZnO (4.84) < blend: 1.5% ZnO (5.06) < blend: 1% ZnO (5.13) < blend: 0.5% ZnO (5.25) < GPC blend (5.63) < ascorbic acid (8.30). A histogram of the IC_50_ values for all the formulations is shown in Fig. 9.

**Table 6:** DPPH free radical scavenging activity (% ± SD) of the GPC composite films at varying concentrations

Compound	200 µg mL^−1^	100 µg mL^−1^	80 µg mL^−1^	60 µg mL^−1^	40 µg mL^−1^	20 µg mL^−1^	IC_50_ (µg mL^−1^)
GPC blend	97.26 ± 0.91	94.67 ± 1.03	93.43 ± 0.96	91.42 ± 1.11	87.66 ± 1.20	78.03 ± 1.34	5.63
Blend: 0.5% ZnO	97.44 ± 0.80	95.01 ± 0.92	93.84 ± 0.87	91.95 ± 0.99	88.40 ± 1.08	79.21 ± 1.22	5.25
Blend: 1% ZnO	97.50 ± 0.77	95.12 ± 0.89	93.97 ± 0.84	92.12 ± 0.97	88.63 ± 1.05	79.59 ± 1.19	5.13
Blend: 1.5% ZnO	97.53 ± 0.82	95.18 ± 0.95	94.05 ± 0.88	92.22 ± 1.05	88.77 ± 1.12	79.81 ± 1.26	5.06
Blend: 2% ZnO	97.70 ± 0.70	95.50 ± 0.81	94.44 ± 0.78	92.72 ± 0.90	89.47 ± 0.97	80.94 ± 1.10	4.71
Blend: 2.5% ZnO	97.64 ± 0.74	95.38 ± 0.86	94.30 ± 0.82	92.54 ± 0.94	89.21 ± 1.01	80.52 ± 1.15	4.84
Ascorbic acid	96.02 ± 0.50	92.34 ± 0.56	90.60 ± 0.60	87.85 ± 0.65	82.82 ± 0.72	70.67 ± 0.85	8.30

This strong antioxidant activity, especially for 2% ZnO, with an IC_50_ of 4.71 µg mL^−1^, was attributed to the antioxidant property of chitosan and the redox activity of ZnO-NPs. The non-monotonic evolution of the IC_50_ value with the increase in the concentration of ZnO indicates that 2.0% loading of ZnO provides optimal interface interaction between the polymer and the NPs, with the most surface-exposed antioxidant functional groups, whereas at 2.5%, agglomeration of NPs partly buries the active sites. The better antioxidant activity of the composite films than ascorbic acid indicates their pharmaceutical applications, especially as wound dressings where the protection against oxidative stress in the wound microenvironment is desired. The combined results of optimum mechanical, barrier, antibacterial, and antioxidant properties at 2% ZnO doping clearly suggest that this formulation could be selected for further pharmaceutical development, and that the GPC matrix could provide a more multifunctional platform compared to the binary polymer systems.

### Photocatalytic activity

The photocatalytic properties of bare ZnO NPs and the GPC–ZnO nanocomposite were tested by degradation of Direct Green 6 dye (DG-6) after being exposed to visible light. Degradation efficiency was quantified by the progressive shift of the characteristic DG-6 absorption peak at *λ*_max_ ≈ 623 nm, which was recorded over time intervals. Several parameters were investigated. GPC–ZnO was able to degrade DG-6 by nearly 95% in 90 minutes, whereas bare ZnO showed only 68% degradation under the same conditions.

### Photocatalytic mechanism and radical scavenger study

Reactive species involved in the degradation of DG-6 were confirmed in radical scavenger experiments. Scavenging experiments were performed using isopropanol (IPA, ˙OH scavenger), benzoquinone (BQ, ˙O_2_^−^ scavenger), and ethylenediaminetetraacetic acid disodium salt (EDTA–Na_2_, h^+^ scavenger), each at a concentration of 1 mmol L^−1^, to identify the principal reactive species. In the absence of a scavenger, degradation was 95.03% ± 0.40%. Addition of 1 mmol per L BQ decreased degradation to 42.97% ± 0.42%, whereas IPA and EDTA–Na_2_ reduced it to 58.00% ± 0.56% and 73.97% ± 0.71%, respectively. The strongest inhibition by BQ indicates that ˙O_2_^−^ is the dominant reactive species, while ˙OH and h^+^ also contribute. A plausible pathway is therefore:ZnO + *hν* → e^−^(CB) + h^+^(VB), e^−^(CB) + O_2_ → ˙O_2_^−^,andh^+^(VB) + H_2_O/OH^−^ → ˙OH.

These are then the reactive oxygen species that will oxidize DG-6 and its intermediates.^21^ In this work, the GPC matrix is not used as an independent semiconductor but is used as an interfacial environment that can improve dye adsorption and generate charge-trapping pathways capable of reducing electron–hole recombination. These effects are not only optical, interfacial, dispersion and adsorption effects, but rather combined effects, thus making it difficult to determine which is the most important.

### Effect of degradation time

**Fig. 10:**
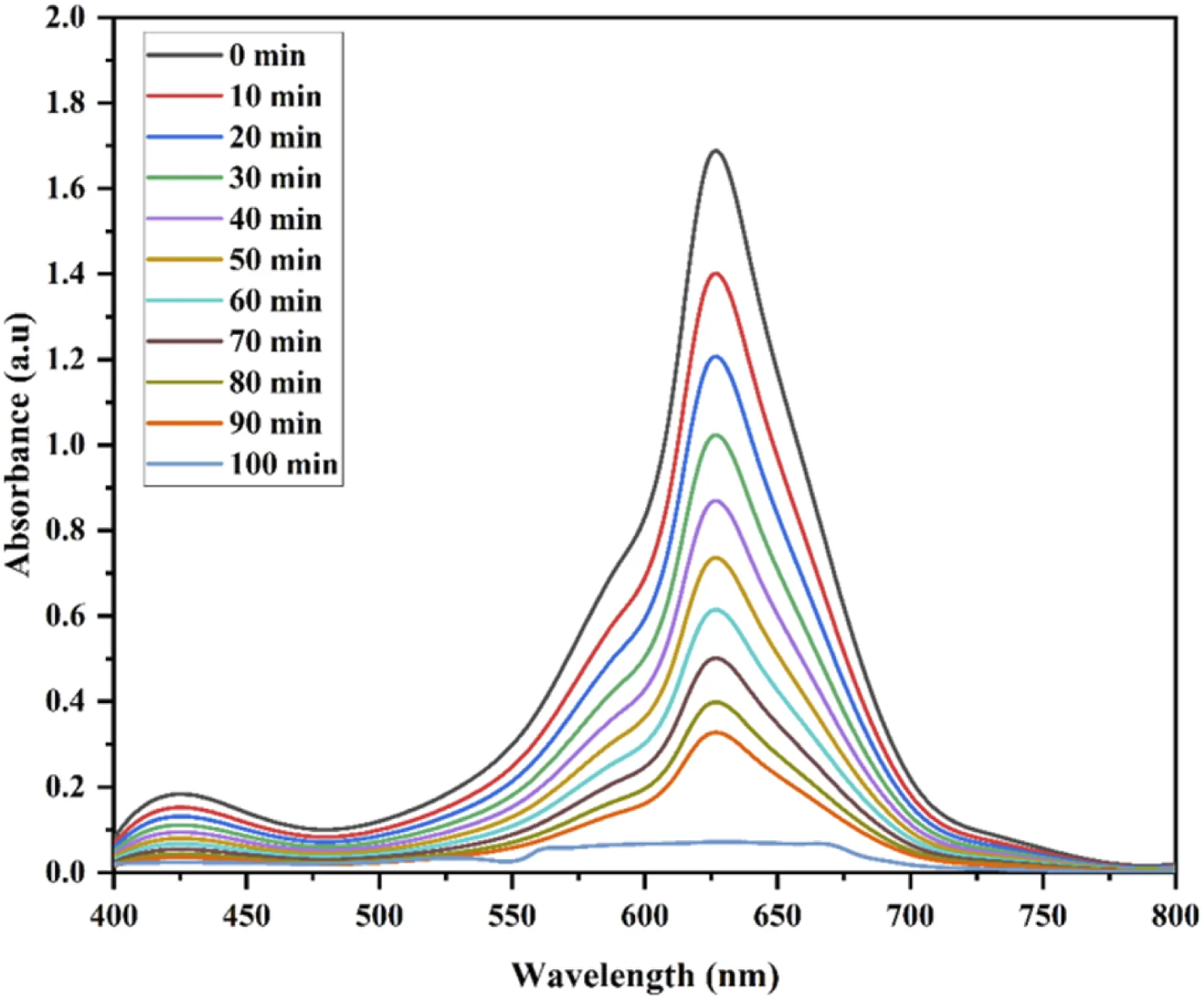
UV-vis absorbance spectra of DG-6 as a function of time over the ZnO nanoparticles under visible-light irradiation.

To study the influence of irradiation time on the photocatalytic degradation, the dye solution was first stirred in the dark for 30 min before being exposed to light to reach an adsorption–desorption equilibrium. After this equilibration period, light irradiation was started. Aliquots (3 mL) were taken at 10 min intervals for up to 90 min. Each sample was centrifuged before quantitative analysis to isolate the photocatalyst. The remaining amount of Direct Green 6 (DG-6) was then measured using UV-vis spectrophotometry at the wavelength of maximum absorption (∼623 nm), as displayed in Fig. 10.

### Effect of photocatalyst concentration

**Fig. 11:**
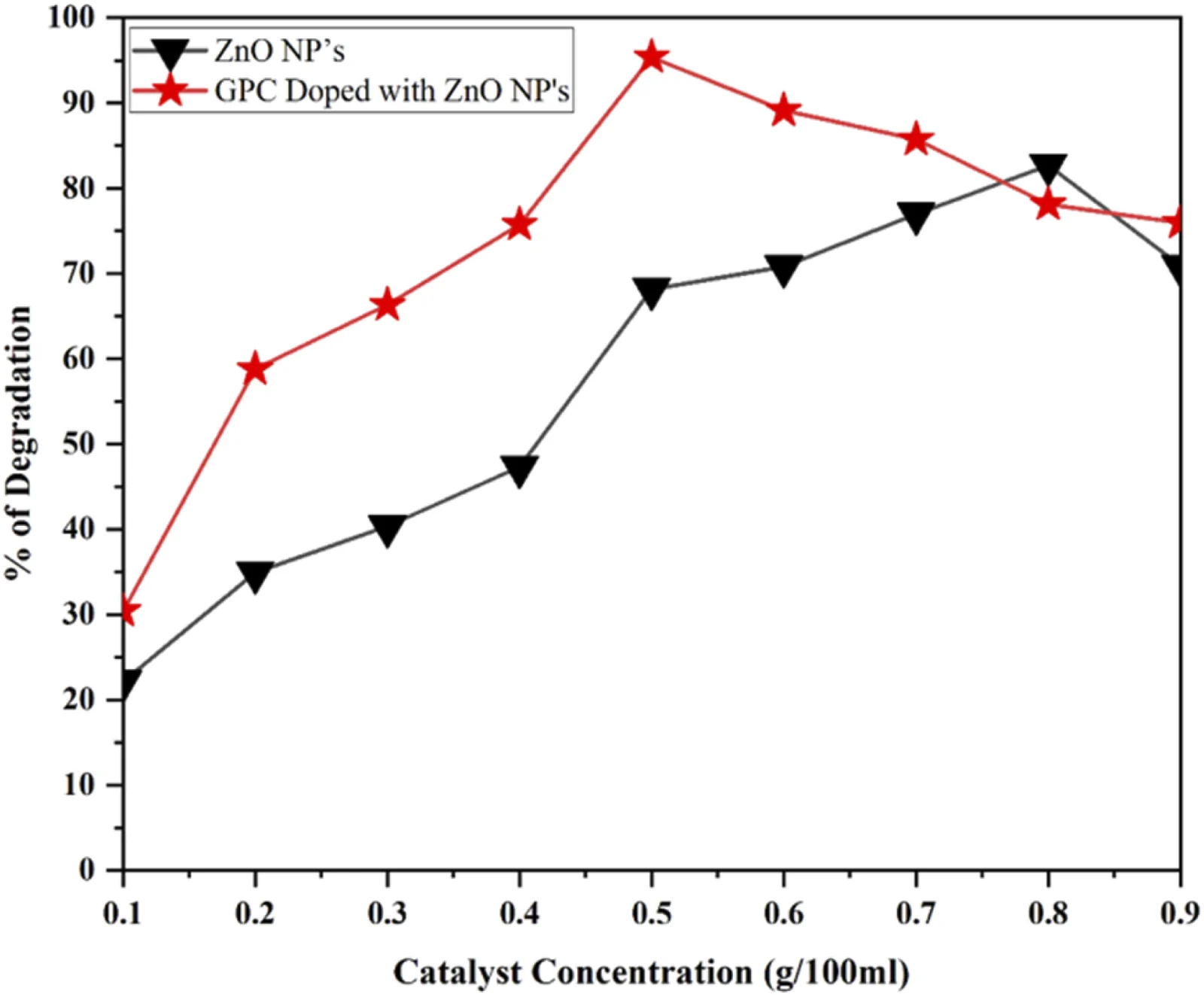
Effect of the concentration of catalyst on DG-6 under visible light irradiation.

The concentration of the catalyst is one of the major parameters of the photocatalytic process that affects the decolorization of dye solutions. In this study, a varying concentration range of the catalyst from 0.1 g to 0.9 g/100 mL was used. As seen in Fig. 11, the degradation efficiency for Direct Green 6 (DG-6) showed an initial increase with the increase in the concentration of both the ZnO-NPs and GPC doped with 2.0% ZnO NPs. However, no significant improvement in degradation was observed beyond 0.5 g/100 mL for GPC–ZnO and 0.8 g/100 mL for bare ZnO NPs, and, in some samples, a slight decrease in efficiency was observed. This decrease is attributed to the aggregation of NPs at high concentrations, which decreases the effective surface area for the absorption of photons and thus decreases the photocatalytic activity of the nanocomposite. At the optimum amount of catalyst (0.5 g/100 mL), degradation of DG-6 was seen to be significantly higher for the GPCs–ZnO nanocomposite (∼95%) than for the bare ZnO (∼68%). Since the degradation efficiency of GPC–ZnO at 0.5 g/100 mL was not significantly different from that at higher dosages, for increased cost-effectiveness, 0.5 g/100 mL of GPC–ZnO was used as the optimal dosage of the catalyst for subsequent experiments.

### Effect of dye concentration

**Fig. 12:**
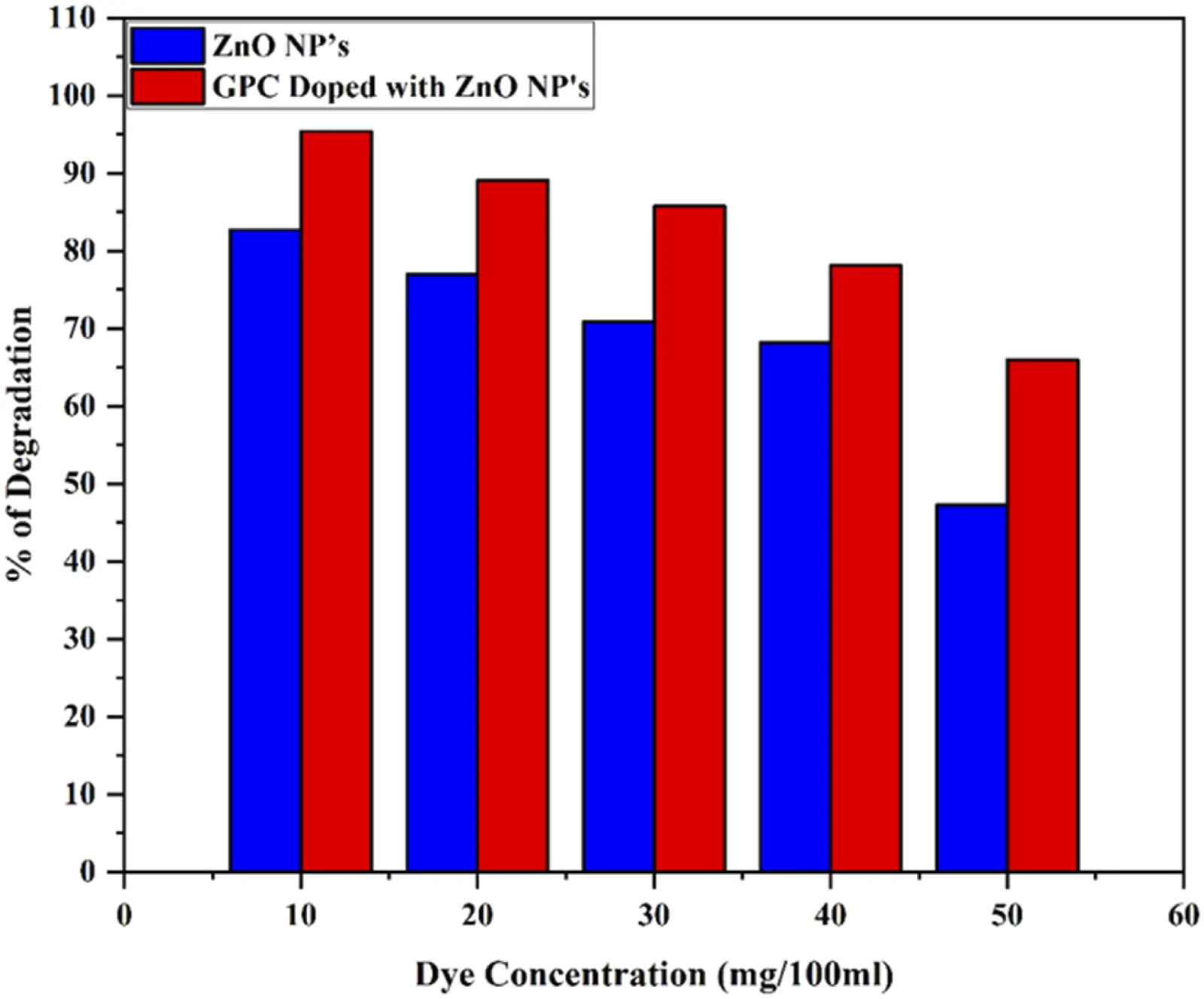
Effect of the concentration of DG-6 under visible-light irradiation.

To study the effect of the concentration of Direct Green 6 dye, the concentration was varied between 10 and 50 mg/100 mL. The results, shown in Fig. 12, show that the higher the concentration of the dye, the lower the degradation efficiency of the photocatalytic degradation for both ZnO and GPC–ZnO. These results corroborate the results of earlier studies.^20^ As the dye concentration rose, the number of intermediate substances formed during the reaction also rose, and their side reactions competed with the decomposition of mother dye molecules.

As the concentration of the dye increases, UV light is more likely to be absorbed by the dye molecules rather than by the photocatalyst (ZnO or GPC–ZnO) particles, which leads to a decrease in the production of electron–hole pairs and hence the photodegradation efficiency. This effect is known as the inner filter effect. Moreover, at high dye loadings, the number of dye molecules and intermediates becomes saturated on the surface of the catalyst, which reduces the interaction between the catalyst and the photons. It was found that the GPC–ZnO nanocomposite generally shows higher degradation efficiency than bare ZnO under all the dye concentrations studied, which demonstrates the higher photocatalytic activity of the GPC–ZnO nanocomposite.

### Effect of pH

**Fig. 13:**
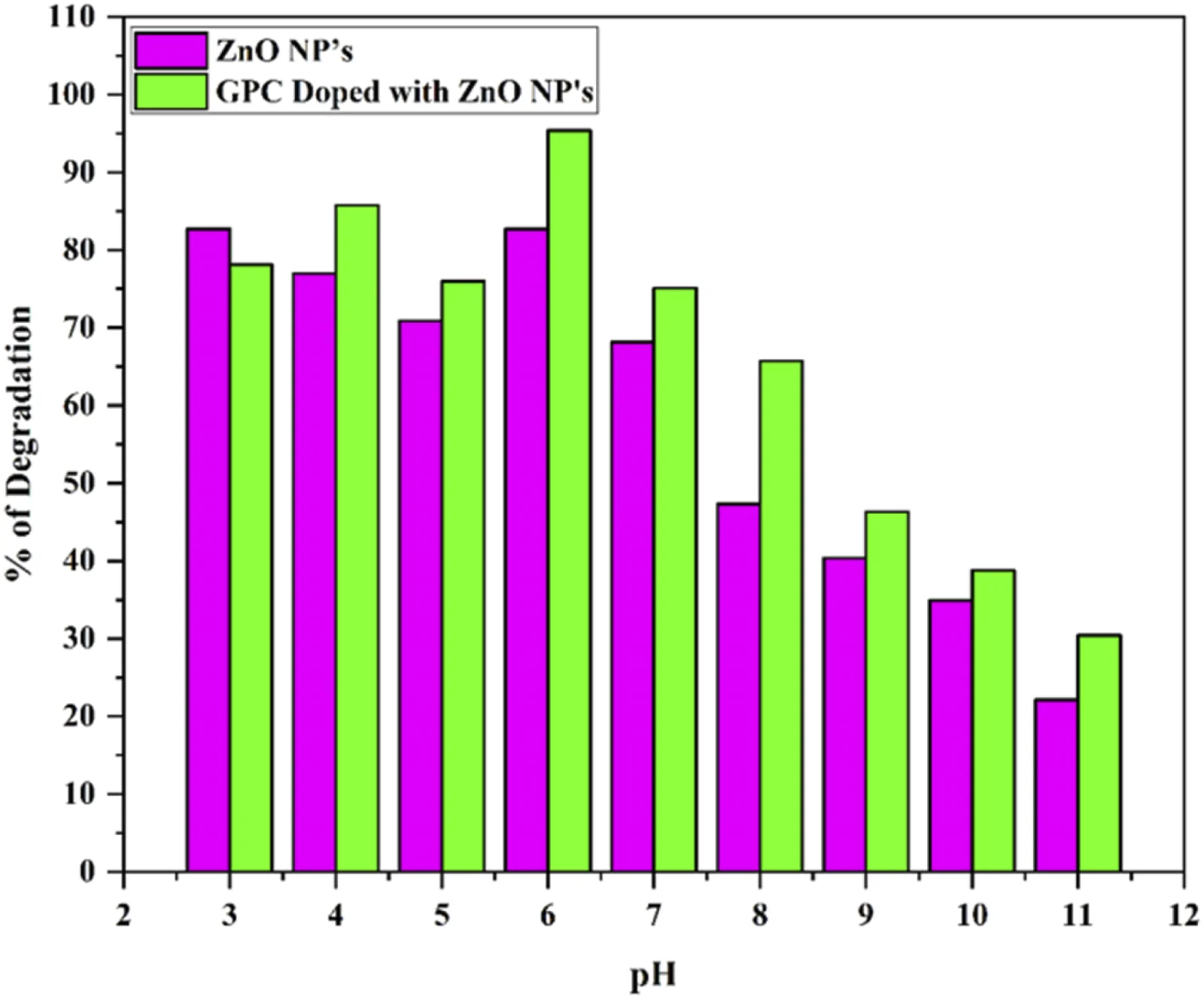
Effect of the pH on the degradation of DG-6 under visible-light irradiation.

The photocatalytic degradation of Direct Green 6 was studied as a function of the solution pH in the range 3–11 at a constant value of the other parameters by using diluted NaOH and HNO_3_ solutions to adjust the pH. The results are shown in Fig. 13. It is seen that the degradation efficiency was maximum at acidic and near-neutral pH (3–6) and decreased gradually at alkaline pH. The photocatalytic process will produce more hydroxyl radicals (˙OH) at lower pH, which will promote photocatalytic degradation, and lower pH will increase the active surface area and contact area between dye molecules and ZnO and GPC–ZnO, resulting in high photocatalytic degradation. The point of zero charge (pH_zpc_) of ZnO is about 9.0; thus, the catalyst surface is positively charged below this value. The presence of sulfonate groups (–SO_3_^−^) in Direct Green 6, as an anionic direct dye, and the electrostatic attraction force between the positively charged catalyst surface and the anions of Direct Green 6 increased at lower pH values, which is beneficial for the adsorption of the Direct Green 6 dye and, therefore, the photocatalytic activity. The degradation efficiency for both catalysts was found to be lower in the strongly alkaline pH range (9–11), which may be due to the partial dissolution of ZnO at high pH, the anionic charge of the catalyst surface repelling anionic dye species, and the scavenging of ˙OH radicals by the excess amount of OH^−^ ions present in the solution. It was observed that over the studied pH range, the efficiency of degradation on the GPC–ZnO nanocomposite was greater than that of bare ZnO, and the optimum pH for both the catalysts was found to be in the range of 5–7.

### Effect of temperature

The reaction temperature was changed from 30–50 °C at a fixed value of all other parameters to assess the influence of temperature on photocatalytic degradation of the dye. The degradation efficiency of bare ZnO and GPC–ZnO nanocomposite increased gradually with the increase in the reaction temperature. As the temperature rises, the bubbles generated in the reaction solution also increase, which facilitates the generation of free radicals and the mass transfer process of the reactants to the catalyst surface. Moreover, the higher temperature enhances the photocatalytic reaction by allowing it to compete more efficiently against recombination of the electron–holes as the higher temperature would give more activation energy to the charge carriers. The higher temperature also improves the rate of oxidation of Direct Green 6 at the catalyst–solution interface. These combined effects lead to a progressive increase in the degradation efficiency as the temperature increases. In addition, GPC–ZnO always yielded higher efficiency at each temperature than bare ZnO, and the efficiency with temperature is more significant for GPC–ZnO, indicating that the synergistic interactions and thermal stability of the GPC matrix further promote charge-transfer kinetics at higher temperatures.

## Conclusions

GPC composite films with 0.5–2.5% (w/w) ZnO-NPs were successfully developed by solution casting, and their physicochemical, biological and photocatalytic properties were evaluated. FT-IR and XRD studies suggested strong interactions and retention of the wurtzite phase of ZnO with the polymers, whereas the Scherrer analysis yielded an average crystallite size of ∼10.20 nm. The direct optical band gap of GPC–ZnO with 2.5% ZnO was slightly lower than that of bare ZnO as determined by the UV-vis DRS, which was 3.18 eV compared to 3.24 eV for bare ZnO. SEM analysis revealed good dispersion at lower and optimal loadings, and localized cracking and defect sizes were observed at 2.5% ZnO. The 2% ZnO formulation showed the most favorable mechanical strength, water-barrier performance, antibacterial and DPPH antioxidant response. The results indicate that the GPC–ZnO achieved around 95% degradation of DG-6 under optimized photocatalytic conditions, while the bare ZnO-NPs achieved about 68% degradation of DG-6 under the optimized photocatalytic conditions. A series of radical-scavenger experiments showed that the predominant reactive species is ˙O_2_^−^, followed by ˙OH and h^+^. The increased performance is attributed to the combined effect of interfacial polymer–ZnO interactions, improved dispersion of nanoparticles and adsorption of dye, limited optical-band-gap modification and better charge utilization. The results presented here demonstrate that further development of the optimized GPC–ZnO film is possible for future use in wound-dressing applications, as well as in antimicrobial barrier applications, environmental photocatalysis applications and packaging applications of pharmaceuticals. Further research should include *in vitro* cytotoxicity studies, controlled release of drugs, *in vivo* wound-healing evaluation and photocatalytic recyclability studies to further validate its comprehensive applicability for industrial and biomedical translation of these nanocomposite films.

## Author contributions

Maqam Ali: conceptualization, experimental work, data collection, and manuscript drafting. Muhammad Kaleem Khosa: supervision, methodology design, data interpretation, and manuscript review and editing. Awal Noor: funding acquisition and manuscript review and editing. Sadaf Qayyum: manuscript review and editing.

## Conflicts of interest

There are no conflicts to declare.

## Data Availability

The original contributions presented in the study are included in the article; further inquiries can be directed to the corresponding authors.
